# Bilateral Mesenchymal Hamartoma of the Chest Wall in a 3-Month-Old Boy: A Case Report and Review of the Literature

**DOI:** 10.1155/2017/2876342

**Published:** 2017-08-16

**Authors:** Mona Alfaraidi, Hossam Alaradati, Irfan Mamoun, Shamayel Mohammed

**Affiliations:** ^1^Department of Pathology and Laboratory Medicine (MBC-10), King Faisal Specialist Hospital and Research Center (KFSH&RC), P.O. Box 3354, Riyadh 11211, Saudi Arabia; ^2^Department of Pathology and Laboratory Medicine, King Faisal Specialist Hospital and Research Center (KFSH&RC), Jeddah, Saudi Arabia; ^3^Department of Radiology, King Faisal Specialist Hospital and Research Center (KFSH&RC), Jeddah, Saudi Arabia

## Abstract

Mesenchymal hamartoma of the chest wall is a well-recognized but extremely rare entity. This entity is believed to be benign with no propensity for invasion or metastasis. Although the lesion manifests with alarming aggressive clinical, radiological, and histological features, it is considered benign and carries an excellent outcome. Therefore it is important to recognize this benign entity to avoid the possible misdiagnosis of malignancy and the unnecessary use of chemotherapy. We present a case of bilateral multifocal mesenchymal hamartomas of the chest wall in a male infant and a literature review of this entity. Our aim is to improve the awareness of this condition and highlight its benign behavior and satisfactory outcome following complete surgical resection.

## 1. Introduction

Mesenchymal hamartoma of the chest wall (MHCW) is a rare benign lesion with an incidence estimated to be 1 in 3000 among primary bone tumors and less than 1 in a million within the general population [1]. MHCW is often misdiagnosed as a malignant tumor due to its dramatic clinical presentation and microscopic features which might overlap with malignant soft tissue or bone tumors. The lesion is thought to develop in the antenatal period and presents usually in neonates and infants. Typically, it arises from one or more adjoining ribs and occurs as a unilateral solitary lesion in the majority of cases. It presents, very rarely, as bilateral lesions. In this report we describe a case of bilateral multifocal MHCW in a boy infant.

## 2. Case Report

### 2.1. Clinical History

A 3-month-old boy presented with respiratory distress associated with bilateral chest masses and deformities, mainly, of the right chest wall. On physical examination, the right chest mass was larger in size than the left mass and was measuring 6 cm. Breath sounds on the right side of the thorax were markedly decreased. On admission, the patient was in mild respiratory distress on face mask oxygen. However, over the following forty-eight hours, his clinical condition deteriorated and was shifted to the intensive care unit where he was intubated and mechanically ventilated.

### 2.2. Radiologic Findings

**Figure 1:**
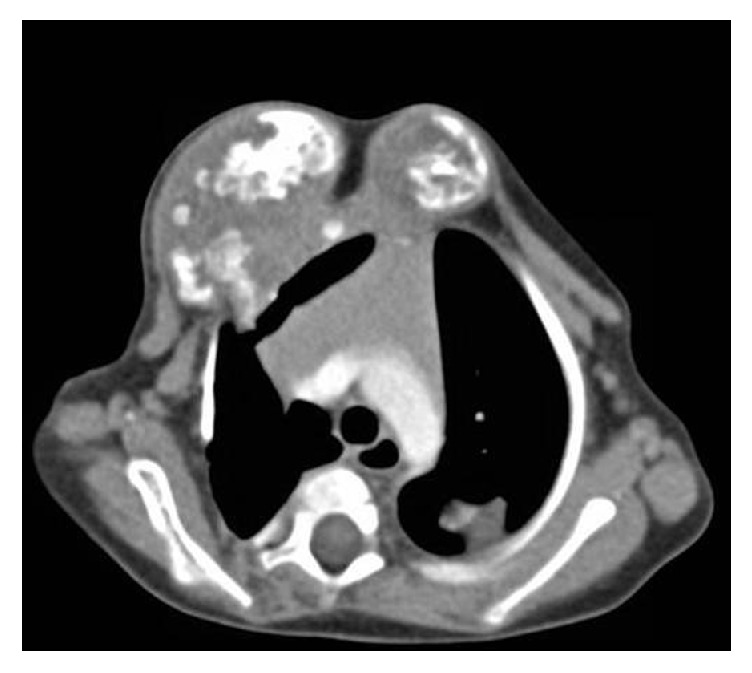
This contrast chest CT scan shows multiple bilateral masses involving mainly the upper ribs, extending into the thorax and causing mediastinal shift to the left. The largest mass measuring 5,7 cm × 4,1 cm is located on the right side. The masses consist of large multiloculated soft tissue lesions with calcifications. The appearance is mostly suggestive of a chest wall bone tumor with likely soft tissue metastasis.

Chest X-ray, on admission, showed bilateral expansile lytic lesions with bony and soft tissue components deforming the adjacent thoracic cage and shifting the trachea to the left. Subsequently, a contrast CT scan of the chest was done and revealed multiple bilateral masses arising from the anterior chest wall, bilaterally (Figure 1). The largest mass was located on the right side of the midline and measured 5,7 cm × 4,1 cm. The masses were involving the upper ribs on both sides and were extending into the intercostal spaces. They consisted of multiloculated soft tissue lesions with areas of calcification causing significant mediastinal shift to the left side and destruction of the surrounding ribs. The overall radiological impression was that of a chest wall bone tumor extending to the hemithorax with suspicious metastatic soft tissue lesions.

### 2.3. Pathologic Findings

**Figure 2:**
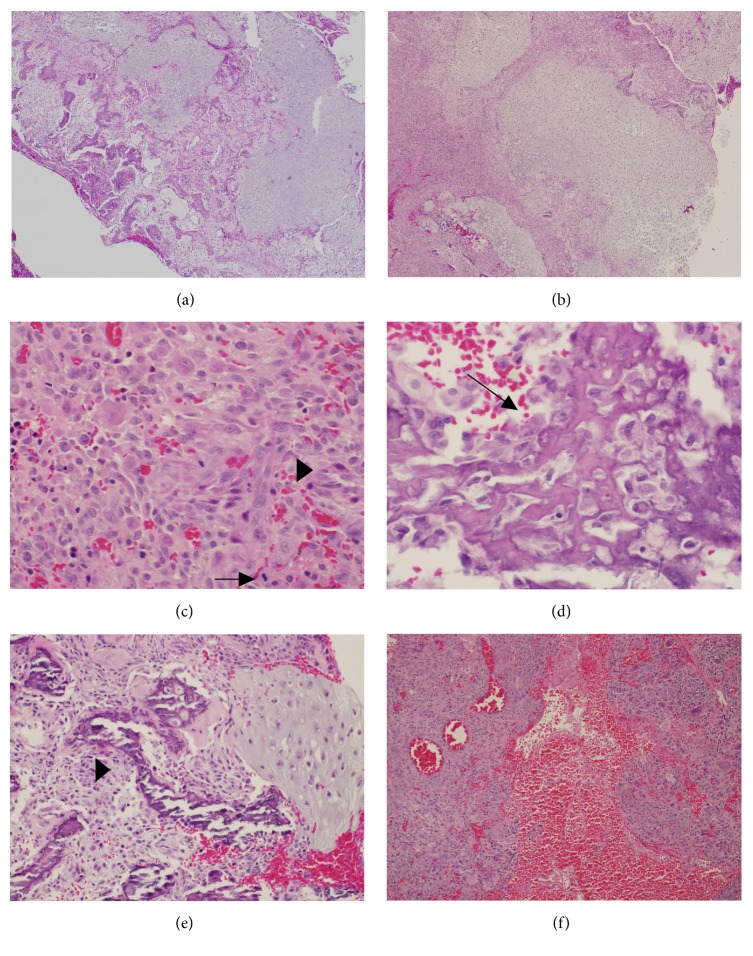
Microscopic findings: (a + b) islands of hypercellular cartilage interspersed within mesenchymal-like stroma of spindle cells. (c) Magnified image of the mesenchymal stroma showing mild atypia (arrow head) and mitosis (arrow). (d + e) Magnified image showing focal endochondral ossification (arrow head) and bone trabeculae (arrow). (f) Area of aneurysmal bone cyst formed of hemorrhagic dilated cystic spaces lined by osteoclasts, giant cells, and collagenous tissue.

After stabilizing the patient, an incisional biopsy was performed. Grossly, the specimen consisted of multiple fragments of a mixture of soft and hard tissues. The fragments had a hemorrhagic tan colored surface and measured 3 cm × 2 cm × 1 cm. Microscopic examination revealed solid areas composed of islands of multilobulated hypercellular hyaline cartilage interspersed within a mesenchymal-like stroma of bland looking spindle cells (Figures 2(a) and 2(b)). Occasional mitotic activity and mild cellular atypia were identified (Figure 2(c)). Around the chondroid tissue, focal endochondral ossification and trabeculae of bone formation mimicking osteoid formation were noted (Figures 2(d) and 2(e)). In addition, there were areas of aneurysmal bone cyst changes formed of hemorrhagic dilated cystic spaces (Figure 2(f)) and the possibility of osteosarcoma was raised on the frozen section. However, after careful examination of the permanent sections, it was noted that the chondroid areas consisted of benign hypercellular cartilage. No malignant osteoid was identified. The constellation of findings of benign hypercellular cartilage with focal endochondral ossification and trabeculae of reactive bone formation with intermixed bland spindle cells along with areas of aneurysmal bone cyst formation and fragments of adipose tissue were characteristic of mesenchymal hamartoma of the chest wall.

Since a malignant bone tumor was suspected initially on the basis of the clinical, radiological, and pathologic findings, starting the patient on chemotherapy was planned. However, on further extensive review of the pathology, the diagnosis of MHCW was confirmed and the patient underwent complete surgical removal of the largest right side mass.

### 2.4. Postoperative Course

The patient had an uneventful recovery and was discharged in a stable condition on the 5th day postoperatively. He will require future clinical follow-up appointments and radiograph evaluations to assess and monitor the remaining masses. He is currently 14 months old, doing fine, and growing well with mild scoliosis in the chest region.

## 3. Discussion

Mesenchymal hamartomas of the chest wall is a rare, benign lesion of uncertain pathogenesis presenting mainly in the neonatal period or in early infancy. Such lesions were described in early reports as osteochondroma, osteochondrosarcoma, and malignant mesenchymoma until the term mesenchymal hamartoma was proposed in 1979 by McLeod and Dahlin [2]. The term mesenchymal hamartoma is now considered the most appropriate term, as it best reflects the benign nature and multiple histological components of the this lesion [3]. Odell and Benjamin were the first to use the term “mesenchymal hamartoma of the chest wall” in 1986 [4].

The presentation of these lesions at birth favors an embryological and developmental pathogenesis over a neoplastic one [5]. Typically, MHCW arise as solitary lesions from the central portions of one or several ribs. The size of the tumor can range from few centimeters to a very large mass [6]. The lesion may progressively enlarge and extend in to the extrapleural space distorting the lungs and mediastinum and filling most of the thoracic cage [7]. Mesenchymal hamartoma of the chest wall are usually unilateral and commonly seen on the right side with a male-to-female ratio of 2 : 1 [2, 3]. Review of the literature revealed ten bilateral reported cases [2, 3, 6, 8]. Our case is one of the unusual presentations with multiple bilateral lesions.

Depending on the size of the tumor and the resulting mass effect on the lungs and mediastinal structures, the clinical presentation of MHCW ranges from an asymptomatic patient to a patient with severe respiratory distress. Chest wall masses and respiratory compromise are the most commonly presenting symptoms. Less common manifestations are scoliosis, chest wall deformity, cough, and fever. Despite the benign nature of MHCW, respiratory compromise caused by extension of the lesion into the thorax may be so severe causing death [3, 9].

One hundred and four cases have been identified in the literature. The reported mesenchymal hamartomas of the chest were mostly nonfamilial except for two cases detected in siblings [10]. In the majority of cases, the lesions occurred in isolation, with no associated congenital anomalies. However, one case has been described in a child with Beckwith-Wiedemann syndrome [2]. The age at presentation ranged from birth to 16 years old [3], although a case of MHCW in a 60-year-old man has been reported in the literature [11]. Three deaths have been reported in association with MHCW. Two deaths occurred immediately after birth and were due to severe respiratory compromise resulting from compression of the lungs by large masses. The third death occurred in the patient with Beckwith-Wiedemann syndrome [3].

Imaging studies are helpful but not considered diagnostic and may be misleading due to the destructive picture of the lesion mimicking chest wall malignancies. On chest X-ray, MHCW appears as a mass showing variable calcifications and arising from one or more ribs. The involved ribs show expansion and destruction and sometimes cause displacement of adjacent ribs. MHCW might be large enough to compress the underlying lung, and secondary changes of tracheal deviation and scoliosis might develop [4]. The typical appearance of MHCW on CT scan is a large heterogeneous expansile rib lesion appearing as an extrapleural soft tissue mass with areas of calcification and fluid-filled cysts, commonly accompanied with some parenchymal compression and mediastinal shift [12]. The common radiological differential diagnosis includes Ewing sarcoma, primitive neuroectodermal tumor of chest wall also called Askin tumor, and chondrosarcoma of the ribs. The multifocal nature of the tumor can be confused with lymphoma, leukemia, and metastasis from neuroblastoma [11].

A definitive diagnosis can only be made by histopathological examination. Biopsy of the lesion can be complicated by severe bleeding due to disruption of the vascular spaces; therefore, needle biopsy should be performed cautiously [12]. Macroscopically, these lesions are well delineated, often lobulated, and bloody in appearance. The cut surface shows cystic and solid areas [3, 4, 10]. Pathologic examination of MHCW reveals that the lesion is composed of a disorganized mixture of fibrous tissue, dilated blood-filled spaces, cartilage, and bone. There is abundant cartilage with focal endochondral ossification and trabeculae bone formation without malignant osteoid formation. Focal stromal hypercellularity may be present, but abnormal mitoses and atypia are not seen. Areas resembling aneurysmal bone cyst, with osteoclast-like giant cells, blood-filled spaces, hemosiderin-laden macrophages, and fibromembranous septa are often present [6]. With this unique histologic features, immunohistochemistry is generally not necessary to establish the diagnosis and up to this date no molecular genetic studies have been performed on MHCW cases [6, 7].

Variable growth patterns are observed including rapid initial growth followed by slower growth, arrest, or regression [12]. The more common outcome for the lesion is a progressive but benign growth. Spontaneous regression of bilateral MHCW has been documented [3]. There is also one instance of malignant transformation described; however, the pathology in this case has been questioned by subsequent authors [11]. This single case of suspicious malignant transformation was later diagnosed as fibrous hamartoma of infancy, an entity separate from MHCW [12].

There are two treatment modalities for chest wall mesenchymal hamartoma: surgical resection for patients with respiratory distress or conservative treatment for asymptomatic patients. A complete surgical resection has been the main treatment for MHCW, especially in symptomatic patients [1, 13]. A wide en bloc excision of the involved part of the chest wall including the involved ribs, underlying pleura, intercostal muscles, and neurovascular bundles is the treatment of choice. Radiotherapy or chemotherapy has been used in some cases; however, they do not offer any established therapeutic benefits [11]. Cure has been reported in cases with complete removal of the affected ribs [14]. Local recurrence has also been reported and is thought to be the result of incomplete resection [6].

Postoperative complications include scoliosis and gross chest deformity. It is better to avoid resection of the posterior aspect of the rib cage to minimize the risk of developing scoliosis [12]. Treatment of asymptomatic cases is controversial. Both complete resection and conservative management are advocated [3]. Previous observations of spontaneous regression of the tumors justify managing asymptomatic patients conservatively [6]. The prognosis after surgical treatment is excellent, which asserts the benign hamartomatous nature of the lesions [9].

## 4. Conclusion

Mesenchymal hamartoma of the chest wall is a rare but benign condition of infancy. Bilateral presentation is extremely rare and only ten cases of bilateral masses have been reported in the literature. Its behavior could be aggressive causing respiratory compromise. Misinterpretation of the pathological findings can lead to unnecessary aggressive treatment such as chemotherapy. A proper understanding and recognition of this condition is essential in the diagnosis.
